# Lymphatic filariasis in the Democratic Republic of Congo; micro-stratification overlap mapping (MOM) as a prerequisite for control and surveillance

**DOI:** 10.1186/1756-3305-4-178

**Published:** 2011-09-18

**Authors:** Louise A Kelly-Hope, Brent C Thomas, Moses J Bockarie, David H Molyneux

**Affiliations:** 1 Centre for Neglected Tropical Diseases, Liverpool School of Tropical Medicine, Pembroke Place, Liverpool L3 5QA, UK

## Abstract

**Background:**

The Democratic Republic of Congo (DRC) has a significant burden of lymphatic filariasis (LF) caused by the parasite *Wuchereria bancrofti*. A major impediment to the expansion of the LF elimination programme is the risk of serious adverse events (SAEs) associated with the use of ivermectin in areas co-endemic for onchocerciasis and loiasis. It is important to analyse these and other factors, such as soil transmitted helminths (STH) and malaria co-endemicity, which will impact on LF elimination.

**Results:**

We analysed maps of onchocerciasis community-directed treatment with ivermectin (CDTi) from the African Programme for Onchocerciasis Control (APOC); maps of predicted prevalence of *Loa loa*; planned STH control maps of albendazole (and mebendazole) from the Global Atlas of Helminth Infections (GAHI); and bed nets and insecticide treated nets (ITNs) distribution from Demographic and Health Surveys (DHS) as well as published historic data which were incorporated into overlay maps. We developed an approach we designate as micro-stratification overlap mapping (MOM) to identify areas that will assist the implementation of LF elimination in the DRC. The historic data on LF was found through an extensive review of the literature as no recently published information was available.

**Conclusions:**

This paper identifies an approach that takes account of the various factors that will influence not only country strategies, but suggests that country plans will require a finer resolution mapping than usual, before implementation of LF activities can be efficiently deployed. This is because 1) distribution of ivermectin through APOC projects will already have had an impact of LF intensity and prevalence 2) DRC has been up scaling bed net distribution which will impact over time on transmission of *W. bancrofti *and 3) recently available predictive maps of *L. loa *allow higher risk areas to be identified, which allow LF implementation to be initiated with reduced risk where *L. loa *is considered non-endemic. We believe that using the proposed MOM approach is essential for planning the expanded distribution of drugs for LF programmes in countries co-endemic for filarial infections.

## Background

The Democratic Republic of Congo (DRC) is the largest lymphatic filariasis (LF) endemic country in Africa with over 49 million people at risk [1-3]. The challenge of mapping LF in the 2.3 m sq km of inhabited regions is further compounded by co-endemicity with *Loa loa *and the poor road infrastructure in the post-conflict environment [1]. Delimiting *L. loa *endemic areas using a high resolution mapping strategy, is critical in minimising the risk for severe adverse events (SAEs) associated with mass drug administration (MDA) with ivermectin. Nevertheless, scaling up treatment for soil transmitted helminths (STH) with albendazole and widespread distribution of long-lasting insecticidal treated nets (LLINs) will positively impact LF endemicity. Incorporating these factors into an overlap mapping strategy will constitute a valuable prerequisite for LF control and surveillance.

The Global Programme to Eliminate Lymphatic Filariasis (GPELF) initiated in 2000 aims to eliminate the disease as a public health problem by the year 2020. The recent publication of the Progress Report detailing the successes of the programme to date have been recorded, as well as, the Strategic Plan for the next decade of activities [1]. The principle strategy is to interrupt LF transmission with MDA using annual treatment with the drugs ivermectin and albendazole in countries in Africa co-endemic with onchocerciasis, and elsewhere with DEC and albendazole. Africa has a significant burden of LF caused by the parasite *Wuchereria bancrofti *with 35 endemic countries, and a further 6 countries where the process of the verification of the absence of transmission is underway [1-3].

While GPELF is making progress overall [1,4] with significant economic saving being demonstrated [5], Africa remains behind other regions with 16 countries still to start MDA implementation. Many of these countries are conflict/ post-conflict countries, characterised formally as Least Developed Countries (LDCs) and are among the poorest in the world with minimal human and financial resources [6]. These constraints pose problems for the national LF programmes with the potential to severely hinder the 2020 goal of LF elimination globally. The WHO Strategic Plan [1] identifies two priorities for the Africa Region to achieve the expansion of MDA; first to resolve the treatment challenges posed by the co-endemicity of *L. loa *due to concerns associated with SAEs when treatment with ivermectin is given in onchoceriasis programmes [7,8]; second to explore how best to utilise vector control as a supplemental intervention for interrupting transmission, especially in filarial co-endemic areas.

The expansion of LF programmes in Africa will inevitably spread into *L. loa *and *Onchocerca volvulus *endemic areas. Due to the increased risk of SAEs, the current MDA regime of ivermectin and albendazole is not recommended where *W.bancrofti *prevalence overlaps with *L. loa*, or where all three filarial parasites co-exist unless those implementing onchocerciasis control adhere to strict guidelines developed by the Mectizan Donation Programme. However, recent maps on the distribution of *L. loa *produced from the rapid assessment procedure for loiasis (RAPLOA) [8-11], and of Community Directed Treatment with ivermectin (CDTi) produced from rapid epidemiological mapping for onchocerciasis (REMO) [8,12] help identify high risk areas that may require fine scale mapping and/or alternative intervention strategies.

Importantly, the recent *L. loa *and CDTi distribution maps will also highlight the potential synergies and benefits between the LF elimination and onchocerciasis control programmes [1,12]. For example, it has been shown that CDTi treatment over 5-6 years with coverage of 65% or more could significantly reduce the prevalence and intensity of other filarial parasites, mainly *W. bancrofti *even though there appeared to be little impact on the adult worm [13,14]. Similarly, repeated doses of albendazole used in STH programmes could potentially impact on LF prevalence [15]. Conversely, the scale up of LF elimination programmes using ivermectin and albendazole across large regions of Africa could help to reinforce the achievements made by the onchoceriasis programmes by increasing the use of ivermectin in areas where onchocerciasis was not controlled as it was previously defined as hypo-endemic but now referred to as low transmission areas [8]. This will assist in reducing the potential residual human reservoir population of *O. volvulus *that could then cause re-infection in areas that have been cleared of the disease. The widespread distribution of albendazole as a component of LF programmes will also enhance the efforts of STH programmes [16].

Additional benefits may be gained from malaria vector control programmes, especially in Africa where the filarial parasite *W. bancrofti *is transmitted by *Anopheles *species [17,18]. Malaria is commonly controlled using insecticide treated nets (ITNs)/LLINs, which act as baited traps for the mosquito when a person sleeps under it [19]. The use of ITNs/LLINs has shown to be effective in reducing LF prevalence in a *W. bancrofti - L. loa *co-endemic area in Nigeria [20], and in *W. bancrofti *transmission areas in Kenya [21], Uganda [22] and Papua New Guinea [23]. Currently, there are large scale malaria control programmes under way across Africa, which involve the mass distribution of ITNs/LLINs [19]. The rapid expansion of malaria vector control could also significantly impact LF prevalence, and distribution maps of ITNs/LLINs would help national LF programmes assess their utility, especially in countries yet to start MDA and those with *L. loa *endemic areas.

WHO [1] stated "Better tools are needed to assess risk as are guidelines on criteria for including or excluding people from treatment programmes". This paper seeks to address these issues by introducing the concept of micro-stratification overlap mapping (MOM) of filarial infections and effective interventions in Central Africa to define more precisely where and what strategies for LF programmes might be implemented in areas of *L. loa *co-endemicity, whilst also assessing the historic data available on LF in one of the most important countries in Africa facing this problem, the DRC.

The DRC is a priority country for GPELF since it is yet to start MDA for LF elimination, and there are several significant challenges for the national LF programme. First, DRC is considered to have the second highest population at risk of LF in Africa (est. 49, 140, 000) [1]. However, this risk is not well defined as no recent in depth studies appear to have been carried out, and the historic information available is largely from studies undertaken in the pre-independence era. This data suggests that the distribution of *W. bancrofti *is discontinuous [24-26]. Second, *L. loa *is endemic across large remote geographical areas, and closely associated with dense tropical rain forests which favour the vectors *Chrysops sp. dimidiata *and *silacea *[9,27,28]. Third, DRC is one of the poorest countries in the world and has been ravaged by civil unrest and conflict for nearly two decades [6,29]. It is now classified as a post conflict country, but these conflicts have left a scarcity of resources, poor infrastructure and numerous challenges to the health care system. Fourth, there is little vector control in place as an alternative intervention with less than 10% of households owning an ITN reported in 2007 [19,30,31]. These factors coupled with its sheer geographical size of 2.3 m sq km, limited transport networks and the remoteness of dispersed communities, present major challenges for many health programmes.

The aim of this paper is to review and synthesise the current knowledge of the distribution of *W. bancrofti *in the DRC, and factors that will impact on the control and elimination of LF such as loiasis co-endemicity, onchocerciasis control programmes, STH deworming activities and malaria bed net distributions. We thus introduce a new term Micro-stratification Overlap Mapping (MOM), which we suggest is a prerequisite for planning any future LF programmes in countries where there is co-endemic loiasis and indeed other control programmes which impact on LF implementation. For this reason, MOM needs to be widely applied when any Preventive Chemotherapy Programme is planned given the synergistic impact of ivermectin and albendazole used together in LF elimination or separately in onchocerciasis or STH programmes respectively and where there is bed net distribution. This information is essential to optimise the future LF MDA implementation strategy to ensure safety, maximum cost effectiveness as well as impact.

## Methods

### Study location

The DRC (formerly the Congo Free State, Belgian Congo, Congo-Léopoldville, Congo-Kinshasa, and Zaire), is located in Central Africa. The administrative structure has changed since independence in 1960, and is currently divided into 10 provinces and one major city, Kinshasa (8.4 million), the capital city and main urban agglomerate. Other major cities include Lubumbashi, Mbuji-Mayi, Kolwezi, Kisangani and Matadi. The DRC is the second largest country in Africa by area (approx. 2,345,409 km^2^) and the fourth most populous nation in Africa, with a population of nearly 71 million (35% urban population) [31,32].

The country has a short Atlantic coastline (40 km) encompassing the mouth of the Congo River. It straddles the Equator, and has a tropical climate experiencing very high precipitation (up to 2000 mm annually), which helps sustain the Congo rainforest and the extensive Congo River Basin that occupies nearly the entire country [33,34]. The vast central basin is a low-lying plateau, surrounded by mountains in the east where the climate is cooler and wetter, and mountains in the south where the climate is cooler and drier. Dense tropical rain forest covers the central river basin and eastern highlands.

### LF distribution

To review and synthesise the current knowledge of the distribution of LF, a systematic search for data in peer-reviewed published literature, and national reports was carried out using PubMed, JSTOR, Google, SCOPUS and other online scientific and historical databases. Studies and reports with data on the prevalence of LF infection (as measured by microfilaria, and immunochromatographic card tests- ICTs), disease cases, as well as potential mosquito vectors were identified, and information on the location (province, district, place), and time period (month, year, decade) and prevalence was collated into a database for mapping and descriptive analyses.

The locations were geo-referenced using administrative boundary maps at provincial and district level, and where specific places were reported, the latitude and longitude coordinates were obtained from data available at the GEOnet Names Server [35]. All data were imported into the geographical information system software ArcGIS 9.3 (ESRI, Redlands CA) to produce historical LF and vector distribution maps.

### Loiasis co-endemicity

To examine the potential extent of LF and loiasis co-endemicity, the recent map on the distribution of *L. loa *produced from RAPLOA surveys carried out between 2004 and 2010 across DRC [9,27] was used for comparisons with LF distribution. The *L. loa *map was imported into ArcGIS, and four levels of *L.loa *prevalence (< 5%, 5-20%, 20-40% > 40%) were digitised (i.e. outlined and shaded) based on the interpolated boundaries contained in the map. The different levels of risk were highlighted, specifically the areas with *L.loa *prevalence > 40%, which are associated with an increased risk of SAEs.

In addition, historical maps on *L.loa *and *Chrysops *vector distributions were reviewed as they are likely to be comparable with the LF distribution map. A systematic search for maps and review articles in peer-reviewed published literature, and national reports was carried out. Maps and geo-referenced data were imported into ArcGIS and digitised to produce historical *L.loa *and vector distribution maps for comparison with the current *L.loa *distribution map, and with the historical LF distribution maps.

### Interventions (risks and benefits thereof)

#### Onchocerciasis control programmes

To examine the distribution of ivermectin, and the potential risks associated with filarial co-endemicity, and potential benefits of the onchocerciasis control programme, the CDTi map produced from REMO surveys carried out in 2004 and 2005 [36] was imported into ArcGIS. The CDTi priority areas (i.e. ivermectin treatment areas) were digitised and areas of potential SAEs risk highlighted by overlapping CDTi priority areas with potential LF and high loiasis (> 40%) distributions. The areas that could benefit from ivermectin treatment were highlighted by overlapping CDTi priority areas with potential LF areas and low to average loiasis (< 40%) distributions.

#### Soil Transmitted Helminth (STH) planning

To examine the potential benefits of albendazole (or mebendazole) distribution in potential LF areas, the controlled planning map for STHs in DRC outlining recommended intervention districts of either once yearly mass treatment or twice yearly mass treatment [16] were imported and digitised in ArcGIS. The maps are based on predictive models from a small number of surveys so have considerable limitations, however, they provide a guide of albendazole (or mebendazole) priority areas for future mass treatment campaigns. Currently, the extent of treatment for STHs across DRC is not known. However, the areas that may or may not benefit from a STH programme were highlighted by overlapping treatment areas with potential LF, low risk loiasis and CDTi priority areas.

#### Malaria bed net distributions

To examine the potential impact of vector control on the LF and filarial co-endemic areas, a distribution map on the proportion of households with children under 5 years old owning an ITN [30] produced from cluster data from a Demographic and Health Survey (DHS) carried out in 2007 [31] was imported into ArcGIS. The map was based on 5,524 households from 300 clusters across DRC with ownership ranging from 0 to 72.3%. Based on a midpoint of 35%, two levels of ITN ownership were digitised to highlight low (< 35%) and average to high (> 35%) areas.

The DHS geo-referenced data were examined further to determined the distribution of households owning any bed net (untreated and/or insecticide treated) by cluster, and to examine differences in ownership by Province [31]. The potential risks and benefits of low (< 35%), and average to high (> 35%) bed net and ITN ownership, were highlighted by overlapping the digitised maps and clusters with LF and filarial co-endemic areas.

## Results

### LF distribution

There are several reviews available on the distribution of LF [24-26,37,38], which outline sero-prevalence, clinical and entomological studies or short/case reports carried out in DRC. In total 11 studies/reports describing LF infection or clinical disease were identified and the main findings are summarised in Table 1. Most studies were carried out between the 1930s and 1970s. No study has been published since 1974. The earliest report of LF is from Boma in the Bas Congo Province in 1899, when microfilaria from *Filaria nocturna *(*W. bancrofti*) was found in night blood of a teenage boy [26,39].

**Table 1:** Chronological list of historical studies and reports of *W.bancrofti *prevalence and disease cases

Time	Province	Region/location	Prevalence/cases	Notes	Reference(s)
1900s	Bas Congo	Boma	1 case	*Filaria nocturna *(*W. bancrofti*) found in night blood from a boy	Van Campenhout and Dreypondt 1901 [39]

1930s	Oriental	Uele	Cases	Elephantiasis and hydrocele s - no *W. bancrofti *microfilaria detected	Van den Berghe 1941 [42]

	Bas Congo	Matadi Thysville and Luozi Kinshasa environs (Leopoldville)	12-36% 2-8% 0.1%	42 cases from villages around mouth/lower Congo River 1-4 cases from villages northward from mouth of Congo River 1 case from 1101 individuals examined from villages in region	Henrard et al. 1946 [44]

		Kinshasa Matadi	0.8% 3%	14 cases from 1824 hospital patients screened for infection 47 cases from 1500 hospital patients, villages close to Angola border	Hernard et al. 1946 [44]

	Bandundu	Idiofa Bandundu (Banningville)	3- 16% 3-31%	14 cases from villages along Kasai River 11 cases, most from villages along Kwango River	Henrard et al. 1946 [44]

	Équateur	Mbandaka (Coquilhatville)	1-3%	3-4 cases, all considered to be from external endemic areas	Henrard et al. 1946 [44].

1940s	Bandundu	Kwango River Kwilu River Inzia, Kasai and Wamba Rivers	20% 12% 2.4 - 3.4%	Overall villages on rivers had higher mf (12.8%) rates compared with villages more than 2 km away (1.5%). Villages along the Kwango river had the highest rates.	Fain 1947 [47]

	Bandundu	Kwango River	Cases	Clinical case examination of patients from villages along river	Fain 1951 [49]

	Équateur	Mbandaka (Coquilhatville)	4.8%	249 prisoners examined, cases likely from external endemic areas	Chardome & Peel 1949 [43]

	Orientale	Yahuma, Basoko District	46%	25 cases from 54 adult males examined in villages south of Yahuma	Bellefontaine 1949 [51]

1950s	Bandundu	Kasongo Lunda	12 cases	Typical signs detected in patients during 188 hernia operations	Van Oye and Pierquin 1961 [38]

	Équateur	Bumba, Banzyville, Gemena, Ikela		Reported to be widespread in northern region of province	Van Oye and Pierquin 1960 [37]

1960s	Oriental	Lomami	63-67% adults	9% in youths, 4% children, cases found in villages along Congo River. Hydrocele in 20% of adult males, low prevalence of elephantiasis	Browne 1960 [50]

	Kasia, Oriental	Not specified	-	Disease widespread, most cases reported from Kasai and Oriental	Dept of Health 1965

	Équateur	Tshuapa (Cuvette Central)	0.6%	4 cases from villages on Maringa River and Lomela River, most villages examined in region were free from disease	Fain et a.l. 1969 [45]

1970s	Bas Congo	Kimbanza, Mayubu region	32%	18 cases from 56 people in one village close to lower Congo River, all other villages in region free of disease.	Fain et al. 1974 [48]

**Figure 1:**
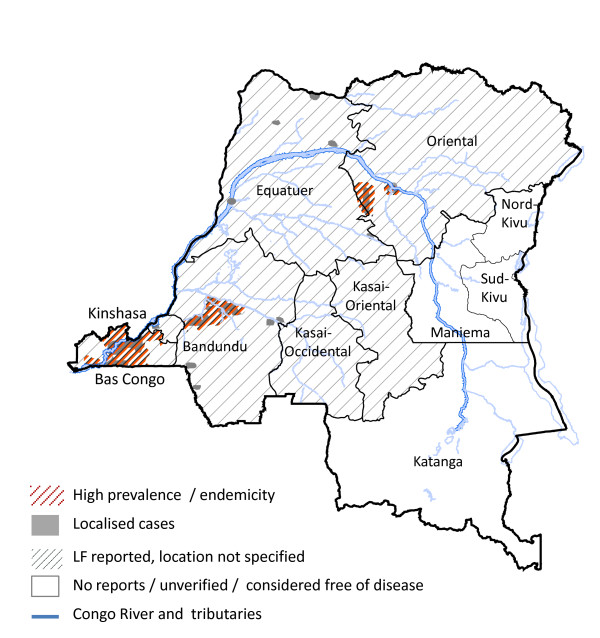
**Historical distribution of reported and suspected/potential *W. bancrofti *prevalence**.

From the limited data available, the prevalence appears to vary within regions and across the country (Figure 1). The review by Sasa [26], cites the absence of *W. bancrofti *in night blood surveys from Pawa, Haut-Uele Province [40], and Yakusa, Oriental Province [41], and highlights that these authors state that *W. bancrofti *was never confirmed with precision from the Congo, and contrary to belief it was considered not to extend across tropical Africa, including the central Congo areas [26,42]. LF was considered not to be prevalent around Mbandaka (Coquilhatville) [43,44] and the region of Tshuapa (Cuvette Centrale) in Équateur Province [45], but is reported to be widespread in northern region close to international borders with South Sudan, Congo Republic and Central African Republic [26,37]. The Department of Health Data report in 1965 [46] reported LF to be widespread with most cases from the Kasai and Oriental Provinces, however, no specific details or locations were provided.

**Figure 2:**
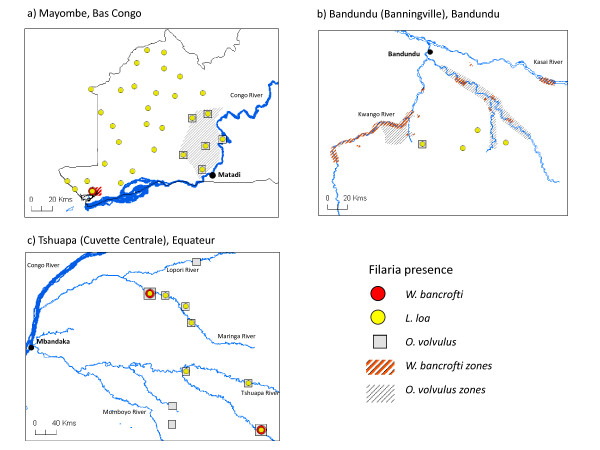
**Micro-stratification of historical data on filarial disease co-endemicity **a) **Mayombe, Bas Congo **b) **Bandundu (Banningville), Bandundu **c) **Tshuapa (Cuvette Centrale), Équateur**.

The most comprehensive studies were carried out in the Bas Congo, Bandundu and Équateur Provinces by Henrard et al. [44], Fain et al. [45] and Fain [47]. The studies in Bas Congo Province showed that the prevalence of *W.bancrofti *varied considerably, with the highest microfilaria (mf) rates reported in villages near the mouth and lower part of the Congo River around Boma and Matadi (12-36%.). Lower rates were found in villages northwards towards Thysville and Luozi (2-8%), and around the Kinshasa region the prevalence was found to be very low or absent (0.1%) [44]. An extensive survey in the forested Mayombe region of Bas Congo found *W. bancrofti *only in one fishing village situated in a swampy area close to the Congo River [48] (Figure 2a).

In Bandundu Province, studies in the Idiofa and Bandundu (Banningville) regions found higher *W. bancrofti *mf rates in villages close to rivers, than those further inland [44,47]. In the Bandundu region, Fain [47] examined 65 villages (2510 individuals) and found that villages along rivers overall had 12.8% mf rates compared with 1.5% among villages more than 2 km away. There were notable differences along and between the different rivers (Table 1) [47], especially the Kwango, Kwilu and Kasai Rivers as shown in Figure 2b. LF prevalence and clinical cases, particularly hydroceles, were also reported in the Kwango River area [49], as well as in other locations close to the Congo River and central basin region [50-52]. In the Tshuapa (Cuvette Central) region of Équateur Province, very few cases were found across the study sites and these were from two villages by the Maringa River and Lomela River [45] (Figure 2c).

Only one study on the vectors of *W. bancrofti *in DRC was found in the literature. This study was carried out by Henrard et al. [44] in Bas Congo in the 1930s. The *Anopheles funestus *was reported to be the most abundant and principal vector in the Matadi and Songololo region with mosquito dissections frequently showing mature larvae, and infection rates up to 4.8%. In the Kinshasa area, *Anopheles gambaie *was considered to be a potential vector, however, *Culex quinquefasciatus (Culex fatigans)*, which was abundant, as to be expected in urban settings, was considered a poor vector [25,44].

### Loiasis co-endemicity

**Figure 3:**
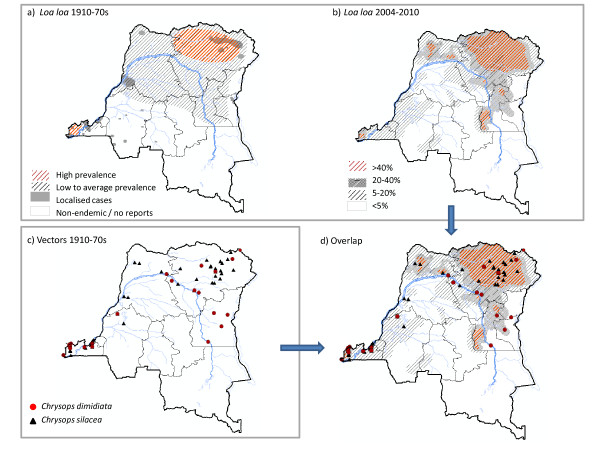
**Distribution of **a) **Loa loa 1910 -70s **b) **Loa loa 2004-2010 **c) **Vectors 1910-1970s **d) **Overlap of Loa loa 2004-2010 and Vectors 1910-70s**.

A map on the historical distribution of *L.loa *in the DRC was produced primarily from maps and data in reviews published the 1960s [28,37,38]. Most data were from studies carried out during the 1930s and 1940s, with additional sites mapped from studies carried out thereafter [26,52]. Figure 3a shows the areas considered to have high prevalence, low to average prevalence, localised cases or no report cases i.e. non-endemic. The highest *L.loa *infections were associated with tropical forest zones and reported in the Mayombe region of Bas Congo Province [48], and the Bas-Uele and Haut-Uele districts of Oriental Province [25,26,28,37,38]. The prevalence in the southern and western provinces was found to be average to low, with some localised foci. The disease was not reported from Katanga and considered to be non-endemic.

The modified map on the current distribution of *L.loa *in the DRC based on the RAPLOA surveys carried out between 2004 and 2010 [9] is shown in Figure 3b. The areas with *L. loa *prevalence > 40% are highlighted to clearly identify areas with an increased risk of SAEs. Similar to the historical map in Figure 3a, the highest rates of infection occurred in the Mayombe region of Bas Congo Province, and in Bas-Uele and Haut-Uele districts of Oriental Province. Additional high risk areas were identified in Maniema Province and the northern region of Équateur Province. Average to low prevalence rates were found in other provinces, and large areas through the central region of the country and in Katanga Province had low i.e. < 5% or no infection.

The distribution of *Chrysops sp *in DRC was most recently reviewed by Fain [28] in the late 1960s. The species *C. silacea *and *C. dimidiata *were identified as the main *L. loa *vectors from collections across the country between 1909 and 1968. In total, *C. silacea *were identified from 52 locations, and *C. dimidiata *from 26 locations, predominantly from the Bas Congo and Oriental Provinces, as shown in the map produced from the available geographical coordinates (Figure 3c). The *C. silacea *and *C. dimidiata *map was overlaid on the current *L.loa *distribution to determine their concordance. Figure 3d shows that most vectors were collected in areas of high i.e. > 40%, or medium i.e. 20-40% prevalence.

The examination of LF and *L. loa *co-endemicity was limited due to the lack of detail and geographical extent of data available. Overall, the LF map and data indicate that high prevalence areas occur in Bas Congo, Bandundu, Équateur and Oriental Provinces, and frequently associated with the Congo River and its tributaries (Table 1; Figures 1, 2a-c). This broadly contrasts to high prevalence *L. loa *distribution, which predominantly occurs in the Oriental Province and areas of Bas Congo, Équateur and Maniema Provinces, and closely associated with tropical dense forests.

On a finer scale, the studies that examined both filaria diseases in sufficient detail [44,45,47,48], suggest that LF and loiasis distributions differ at a micro level. For example, Henrard et al. [44] found more cases of *W. bancrofti *in Matadi, and Bandundu regions than *L. loa*, but more cases of *L.loa *in the Kinshasa, Mbandaka (Coquilhatville), Thysville and Luozi regions than *W. bancrofti*. The reproduction of historical maps and filaria data points from the Mayombe [48], Bandundu (Banningville) [47], and Tshuapa (Cuvette Centrale) [45] further highlight the geographical differences and the localised overlaps between the diseases, (Figures 2a-c). In the forested region of Mayombe and river region of Tshuapa, *L. loa *was most prevalent with only one or two locations reporting both diseases [45,48]. In the river region of Bandundu, *W. bancrofti *was most prevalent in villages bordering the river with no overlap with *L. loa*, which was found in inland villages [47]. Onchocerciasis was also recorded in these studies [45,47,48], and included in the maps to highlight the different co-endemic combinations and the implications of ivermectin treatment.

### Risks and benefits of interventions

**Figure 4:**
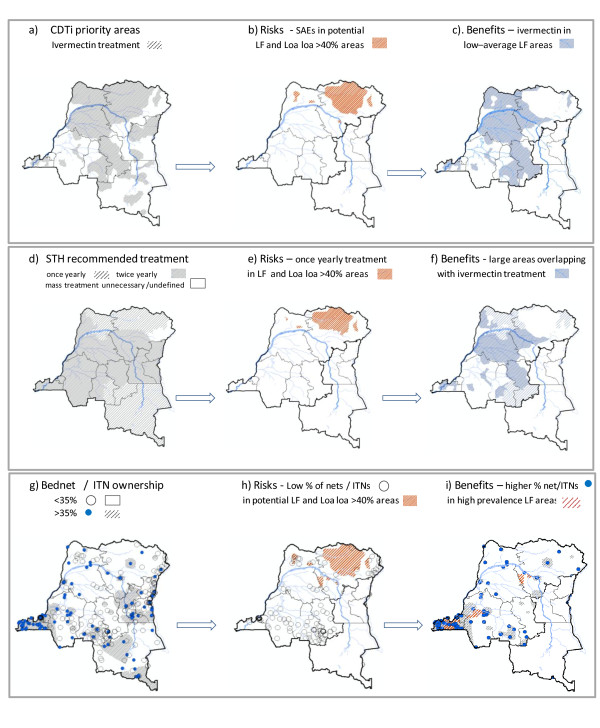
**Interventions distribution maps and associated risks and benefits **a) **CDTi priority areas **b) **Risk of CDTi **c) **benefits of CDTi **d) **STH recommended treatment **e) **Risks of STH treatment **f) **Benefits of STH treatment **g) **Bed net /ITN ownership **h) **Risks of bed net/ITNs **i) **Benefits of bed net /ITNs**.

The onchocerciasis control programme (APOC) CDTi priority areas are shown in Figure 4a[36], and illustrate that large areas in the central and northern region of the country are being targeted with ivermectin treatment. The potential risks associated with ivermectin treatment for *O.volvulus *in potential areas of *W.bancrofti *and high *L.loa *co-endemicity are concentrated in the Oriental Province and areas of the northern Équateur Province (Figure 4b). The potential areas to benefit are extensive and include large areas of Équateur and Kasai-Oriental Provinces and areas of the Oriental, Kasai-Occidental and Bas Congo Provinces (Figure 4c).

The STH recommended treatment areas are shown in Figure 4d[16], and illustrate that most of DRC is recommended for either once yearly or twice yearly albendazole (or mebendazole) treatment. The risks associated with the STHs treatment are minimal but may be related to treatment only recommended once yearly in potential *W.bancrofti *and high *L.loa *co-endemic areas (Figure 4e). The potential benefits of STH treatment are again extensive and include large areas of Équateur and Kasai-Oriental Provinces and areas of the Oriental, Kasai-Occidental and Bas Congo Provinces recommended for twice yearly albendazole (Figure 4f).

The distribution of households owning > 35% ITNs and/or > 35% bed nets (treated or untreated) is shown in Figure 4g[30,31], and illustrates that the highest coverage rates occur in Kinshasa, Bas Congo, Katanga, Maniema, and Sud-Kivu Provinces. The overall percentages for each Province are shown in Table 2[31]. The main risk associated with bed net and ITN distribution is the low coverage in potentially high risk *W.bancrofti *areas such as Bandundu Province, and in areas co-endemic with high *L.loa*, such as the Oriental Province and areas of northern Équateur Province (Figure 4h). The benefits include higher coverage rates in the potentially high risk *W. bancrofti *areas in Bas Congo (Figure 4i).

**Table 2:** Percentage of households owning a mosquito bed net or ITN by Province

Province	Bed net	ITN
Kinshasa	42.2	25.7
Bas-Congo	55.6	42.0
Bandundu	28.2	19.8
Équateur	31.1	9.3
Orientale	13.1	6.8
Nord-Kivu	13.3	8.2
Maniema	32.4	22.3
Sud-Kivu	39.5	17.8
Katanga	34.9	16.7
Kasaï-Oriental	14.2	9.6
Kasaï-Occidental	21.3	13.9

## Discussion

This study highlights the lack of detailed and current data on the distribution of LF in DRC, a country considered to have one of the largest burdens of disease in the world, and second in sub-Saharan Africa, after Nigeria [1]. The national LF Programme in DRC is yet to commence MDA implementation, which is complicated by approximately one third of the country being endemic for loiasis [9]; a contraindication for any MDA regimen which includes ivermectin. The reason for the lack of information and action may be attributed to the widespread civil unrest affecting many parts of the country for decades, which has left it severely under-resourced in public and private sectors, as well as the absence of efficient transports systems making access to many areas of the country difficult [29,32].

The insidious nature of internal instability is a major barrier for disease control programmes, and has implications for the national LF programme in terms of accessing remote, rural endemic regions, readily and safely [32,53]. These problems are not unique to DRC as many of 16 countries still to start MDA in Africa are loiasis endemic and considered to be conflict or post-conflict countries [54]. This group of countries are among the poorest and most fragile in the world [6], which raises the importance of an integrated effort between international partners, and the various national NTD [8,16,36] and vector control programmes [19,55] to ensure that resources are maximised, and the elimination of these diseases achieved collectively.

The review and mapping of historical data in DRC highlights gaps in our knowledge and the difficulties in fully defining the problem. The value of collating and analysing disparate data sources cannot be underestimated. This activity is recommended for countries planning to start MDA, as it will help to identify high risk areas and key risk factors that are crucial to control efforts even if resources are limited. We also advocate that the new mapping approach of MOM is essential in countries where there is co-endemic loiasis as well as other control programmes which impact on LF implementation. However, it may be important to evaluate if transmission is still ongoing in some areas where CDTi or deworming activities have taken place for several years, since reported coverage may not always be true or may not always have the predicted impact on the targeted diseases. Therefore, before excluding an area for LF control, it may be prudent to rapidly assess the impact of other interventions on the LF endemicity.

The simple reproduction and overlapping of historical data and maps, clearly shows the close association of LF distribution with the Congo River and its tributaries in different regions of the country. This provides key information about the ecology and transmission of *W. bancrofti *vectors, which is important in DRC where only one study has been carried out in the past 70 years [44]. In Matadi, Bas Congo, *An. funestus *was identified as the most important vector of *W. bancrofti *[44], however, other entomology studies indicate a diverse range of *Anopheles *across the country [56-58]. An extensive survey found *An. moucheti *and *An. wellcomi *most abundant along the central Congo River system, and *An. paludis *in the surrounding hinterland [59]. In Katanga, the mosquito species were considered to be distinct to other ecological regions [56], and in the forested Mayombe region, Bas Congo, *An. gambaie *s.l was found to be much more abundant than *An. funestus *and *An. moucheti *[60], which may explain the lack of LF found in this region by Fain [48].

The recent maps on the distribution of *L. loa *in Africa [9,27] are a useful resource for the national LF programme in DRC. The *L. loa *maps elucidate SAEs risk areas, where extra precautions and alternative intervention strategies may be required, especially if LF is found to be endemic. Comparisons between the historical and current *L. loa *maps indicate that its distribution has remained relatively stable for more than half a century, and been a long-standing public health problem, particularly in the Oriental Province. The problem extends across large areas of central Africa affecting many vulnerable people [9], and poses a major obstacle to the LF and onchocerciasis elimination programmes in Africa. Whilst *L. loa *is not included in the list of WHO's 17 NTDs [61] its importance as an impediment to progress of programmes based on preventive chemotherapy cannot be understated.

Overlapping the *C. silacea *and *C. dimidiata *distribution data with the *L. loa *map indicates that both vectors may transmit the infection in the high risk areas. In Central Africa these *Chrysops sp *are found to be sympatric in tropical rain forests and rubber plantations, and shown to bite throughout the day and have different annual transmission cycles [26,28]. To eliminate LF in rain forest loiasis co-endemic areas will require new innovative strategies, possibly including *Chrysops *control as a novel approach. Although the development of villages, clearing of vegetation and the spraying of insecticides of *Chrysops *breeding sites have shown to produce a degree of control of loiasis transmission [62-65], these methods are impractical. Therefore, it may better to use impregnated/baited traps or trapping methods similar to those used for Human African Trypansomiasis (HAT), which is transmitted by *Glossina *but shares some biological features of *Chrysops *biting patterns and habitats [66]. Recent data shown in the global atlas of HAT indicates geographical overlap in some high risk *L. loa *areas, and the use of MOM may elucidate areas where both vectors may be trapped for control purposes.

Alternatively, a combination of new drug regimens and integrated vector management (IVM) [67] may be an option in high risk LF areas and co-endemic high loiasis areas. The use of alternative drug strategies such as twice yearly treatment or higher doses of albendazole [15] or 4-6 week course of doxycycline [68] may be a better approach to reduce filaria loads, and reduce the risk of disease and SAEs in selected populations. These alternative drug strategies used in combination with ITN/LLINs and/ or indoor residual spraying (IRS) may also significantly reduce *W. bancrofti *transmission [19]. More synergies between malaria and LF programmes are essential, especially as the distribution of ITN/LLINs is increasing dramatically. Although no large-scale IRS activity is planned for DRC [55], historical IRS activities using gammexane, show considerable reductions in *An. moucheti, An. gambiae *s.l and *An. paludis *densities in DRC [69], and more recently with DDT in *An. funestus *in other countries [70], which suggests that IRS could be targeted with significant impact, especially in Oriental Province. However, it will be important to monitor insecticide resistance of vector control activities [70,71].

The extent to which *W. bancrofti *and *O.volvulus *overlap in *L.loa *> 40% high risk areas in Oriental Province is unknown, however, Woodman [72] in neighbouring southern Sudan illustrates that the three diseases are co-endemic across relatively large geographical areas. The use of MOM could elucidate different patterns of co-endemicity within such areas, to determine if certain interventions would be of risk or benefit. The three finer scale maps in this study show that different regions have different spatial and overlapping patterns with different risks and benefits. For example, in the Mayombe region, Bas Congo [48]*L. loa *was most prevalent, and only overlapped with *W.bancrofti *and *O.volvulus *in distinct areas. This region has high bed net/ITN coverage, which is of significant benefit; however, the overlap of the CDTi priority area in the *L.loa *and *O.volvulus *co-endemic area is a risk as ivermectin treatment could result in SAEs. In contrast, in the Bandundu (Banningville) region [47], the overlaps between the CDTI priority areas and *W. bancrofti *and *O.volvulus *co-endemic areas will be of considerable benefit as ivermectin could reduce the prevalence of both diseases (as well as STHs) along the river with limited risk of SAEs. However, there is a low or no coverage of bed nets/ITNs in this region [30,31].

On a broader scale, the country level maps highlighting the overlaps between different disease distributions and interventions provide important insights into the potential risks and benefits of multiple large-scale disease control programmes operating in one country. It clearly identifies high risk and vulnerable populations which need to be targeted with more effective or alternative innovative intervention strategies. Importantly, it also shows the potential large-scale benefits that the combination of the onchocerciasis, malaria, and future STH programmes could provide to the national LF programme and vice versa. Clearly, there are huge benefits of coordinated and overlapping mapping activities so Ministries of Health have a clear picture of the epidemiology for planning purposes.

## Conclusions

This paper identifies a new approach to mapping that takes account of factors that will influence country LF elimination strategies and plans. The paper suggests that country plans require higher resolution mapping that has been used to date, before implementation of LF activities can be efficiently deployed. This is because 1) distribution of ivermectin through APOC projects will already have had an impact of LF intensity and prevalence 2) DRC as an example, has been up scaling bed net /ITN distribution which will impact over time on transmission of *W. bancrofti *and 3) recently available predictive maps of *L. loa *allow higher risk areas to be identified. This will allow LF implementation to be initiated with reduced risk where *L. loa *is considered non-endemic. We believe that using the proposed MOM approach is a prerequisite for planning the expanded distribution of drugs for LF programmes in countries co-endemic for filarial infections.

## Competing interests

The authors declare that they have no competing interests.

## Authors' contributions

DHM conceived the idea for the study. LKH identified data sources, developed the study design and collated, mapped and analysed the data. BCT assisted with the data collation, mapping and analyses. MJB contributed to the analyses and interpretation of results. All authors contributed to the writing of the manuscript and approved the final version.
